# “The Only People That Really Understand”: A Qualitative Study of Healthcare Workers’ COVID-19 Experiences and Implications for Workplace Support

**DOI:** 10.3390/healthcare14101400

**Published:** 2026-05-20

**Authors:** Brian En Chyi Lee, Elizabeth M. Clancy, Leanne Boyd, Andrea Reupert, Nicholas F. Taylor, Sherrica Senewiratne, Jade Sheen

**Affiliations:** 1 Deakin Lifespan Institute, Faculty of Health, School of Psychology, Deakin University, Geelong, VIC 3220, Australia; 2 Deakin Cyberpsychology Research Group, Deakin Cyber, School of Psychology, Deakin University, Burwood, VIC 3125, Australia; 3 Eastern Health, Melbourne, VIC 3128, Australia; 4 Faculty of Education, Monash University, Melbourne, VIC 3800, Australia; 5 Department of Physiotherapy, Podiatry and Prosthetics and Orthotics, La Trobe University, Melbourne, VIC 3086, Australia; 6 Allied Health Clinical Research Office, Eastern Health, Melbourne, VIC 3128, Australia

**Keywords:** healthcare workers, doctors, nurses, COVID-19, workplace mental health, workforce wellbeing, supervisor support, peer support, healthcare workforce retention

## Abstract

**Highlights:**

**What are the main findings?**

- Demands: Rapid, concurrent workplace change produced cognitive overload, sustained uncertainty, and family-life spillover, with leaders carrying a disproportionate burden.
- Resources: Local management and informal peer support were the most-valued resources, but both were structurally degraded. Leaders absorbed organisational stress at personal cost, and infection-control protocols eroded peer interaction. Formal supports (EAP) were often poorly targeted.

**What are the implications of the main findings?**

- Lessons for today: These dynamics are not pandemic-specific. Strengthening leadership capacity, protecting peer-interaction infrastructure under altered protocols, and embedding work–family-aware policy translate directly to current workforce-sustainability efforts.

**Abstract:**

**Background**: Healthcare systems globally continue to experience persistent workforce and system-level challenges as increased workloads, lasting wellbeing impacts, and retention issues remain following the pandemic. To inform strategies and interventions to address these issues, this paper explored the workplace experiences of Victorian (Australia) frontline healthcare workers with parenting responsibilities during the COVID-19 pandemic. **Methods**: A total of 39 frontline healthcare workers from a large metropolitan hospital were interviewed between October 2020 and February 2021. Reflexive thematic analysis was used to analyse transcripts. **Results**: Three superordinate themes and five subordinate themes were identified. Themes highlighted the significant pressure that rapid workplace changes placed on healthcare staff and leaders, affecting their physical, mental, and relational health. Support from peers and supervisors was protective, though this increased demands on supervisors themselves. While many staff reported pride in their work, some experienced reduced career satisfaction and concerns about lasting psychological impacts. **Conclusions**: This study identifies how workplace supports operate through communication transparency, leadership capacity, and protected peer-support space, translating to organisational priorities for the post-pandemic workforce. In the context of ongoing workforce shortages and heightened demands post-pandemic, these findings underscore the importance of strengthening leadership capacity, embedding sustainable workplace supports, and addressing the psychological needs of healthcare staff. Such system-level responses are essential for pandemic recovery, improving workforce retention and staff wellbeing in the modern healthcare environment.

## 1. Introduction

Healthcare systems globally are currently experiencing significant strain, characterised by workforce shortages, increasing service demand, and growing concerns about workforce sustainability [1,2,3]. While many of these pressures predate the COVID-19 pandemic, the pandemic intensified existing systemic challenges and left a lasting impact on the mental health and wellbeing of healthcare workers (HCWs). Evidence has shown that these ongoing psychological impacts are associated with increased intentions to leave the profession among Australian HCWs [1,3]. Consequently, addressing HCWs’ mental health has become a critical priority for sustaining the healthcare workforce within the Australian healthcare research agenda [2].

Despite many public health efforts during the COVID-19 pandemic, infections and hospitalisations have rapidly surged and surpassed hospital capacities in many countries, placing tremendous strain on healthcare systems worldwide [4,5]. Under these conditions, HCWs, particularly those working in high-risk frontline settings, worked under significant pressure, leading to significant health challenges. Many studies reported that frontline HCWs experienced high rates of mental health and physical issues [6], such as fatigue [7,8], burnout [9,10], depression [11,12], anxiety [10,13], psychological distress [14], post-traumatic stress disorder [12,15,16], and sleep disturbance [16,17]. While some studies suggest that mental health decline among HCWs may have returned to baseline for some [18,19], high work demands and pressures have persisted, and many HCWs continue to report lasting mental health impacts [20,21,22]. This raises important concerns for workforce functioning and retention in modern healthcare systems.

The Job Demands-Resources (JD-R) theory [23,24] suggests that when job demands outweigh the available resources, it can put an employee at risk of experiencing burnout. JD-R extends earlier work-stress frameworks, most notably Karasek’s Job Demand-Control model [25], which proposed that the combination of high psychological demands and low decision latitude is the primary driver of work-related strain. JD-R broadens this by allowing any job demand to be offset by any job resource, rather than restricting the buffering role to control alone.

These job demands can include any type of demand that requires an employee to exert effort. In relation to frontline HCWs, this can include attending to patients, filling out paperwork, or dealing with difficult families. Relevant resources that help frontline HCWs cope with these demands may include sufficient sleep, support from their supervisors and coworkers, and adequate time required to concentrate on their work. Typically, frontline HCWs are resilient when facing challenges in their work, such as dealing with patient illnesses and deaths [26]. However, the COVID-19 pandemic has shown that unique demands and stressors associated with healthcare roles have overwhelmed many frontline HCWs. In addition to their worries around a new coronavirus disease during the pandemic [27,28], frontline HCWs have had to manage a surge in patient care that took place alongside the rapid overhaul in working procedures aimed at preventing nosocomial outbreaks. Balancing [29] these increased workloads with high-quality care during the COVID-19 pandemic has also been confronting for many frontline HCWs who have experienced moral distress as a consequence [29,30].

Furthermore, the pandemic intensified pre-existing pressures on healthcare workers along multiple dimensions, including the social determinants (e.g., housing, income security, systemic inequities, and caring responsibilities [31,32,33]) that shape how individual workers absorb workplace stress. For frontline HCWs living with families, many have had to balance these new work stressors with the increased family responsibilities during school closures and lockdown restrictions [34,35]. This raised concerns about poor work-life balance among frontline HCWs and its impact on their wellbeing. This strain was also heightened by the likelihood of coming into contact with infected patients and potentially putting family members at risk of contracting the virus as a result of this interaction [36]. Another relevant demand identified was the need to quarantine if frontline HCWs were exposed to the virus, which would result in fewer interactions with family and not being able to work [37]. Together, these wide-ranging impacts are concerning, given that a recent study suggests that they were long-lasting, affecting frontline HCWs all the way to the end of the pandemic and potentially post-pandemic [21].

While the pandemic has ended, these impacts on frontline HCWs can be long-lasting and have implications for modern-day healthcare systems. Many studies have documented these impacts of healthcare work during the COVID-19 pandemic on frontline HCWs’ mental health, but it is equally important to deeply understand frontline HCWs’ lived experiences of these stressors. However, lived-experience evidence on how workplace supports operate in practice, not only whether they are used, remains limited, particularly when examined under a JD-R lens, despite its direct relevance for organisational design. These dynamics offer transferable lessons for healthcare systems facing continued workforce strain, climate-driven surge events, and conflict-related disruptions. Documenting and learning from these experiences is therefore essential to inform consumer-led supports and organisational responses that remain effective and relevant for the modern healthcare workforce.

### Aims

This study aimed to explore the impact of working in frontline healthcare roles during the COVID-19 pandemic in a sample of frontline HCWs with parenting responsibilities. The following questions were investigated:

1. What are the work-related experiences (or demands) of frontline HCWs staffing Victorian hospitals during the COVID-19 pandemic?
2. What strategies or supports (i.e., job resources) do frontline HCWs find more or less helpful in supporting their work roles during the COVID-19 pandemic?
3. What lessons from frontline HCWs’ experiences during the COVID-19 pandemic can inform current workplace policies and support systems for healthcare workers?

## 2. Methods

### 2.1. Study Design

This study reports on data collected from a longitudinal qualitative cohort study on frontline HCWs with parenting roles working in Victoria, Australia [38]. Data collection occurred between October 2020 and February 2021 through semi-structured interviews. This study was reported according to the COnsolidated criteria for REporting Qualitative research (COREQ) Checklist [39] (see Supplementary Materials).

### 2.2. Participants and Recruitment

Study participants included 39 frontline healthcare workers employed in metropolitan hospitals in Victoria, Australia. Participants were eligible if they were aged ≥18, employed in a frontline clinical role at a Victorian metropolitan hospital working in a COVID-facing area (Accident and Emergency, COVID wards, Intensive Care, or Hospital in the Home teams), had parenting responsibilities for at least one child aged under 18 at the time of recruitment, were able to complete a semi-structured English-language interview, and provided written informed consent. Participants were primarily recruited through the workplace communications of a large metropolitan health service in Victoria, Australia. Online social media platforms (e.g., Facebook, Instagram, LinkedIn) were also used for recruitment. Adult participant details are presented in Table 1. Participants ranged in age from 29 to 57 years (*M* = 41.6, *SD* = 7.1). They had between one and four children (*M* = 2.1), with 84 children represented in this study (in addition to four pregnancies). All participants in this study were working with confirmed or suspected COVID cases within their healthcare roles. No participants dropped out during data collection.

### 2.3. Procedure

Ethical approval for this study was granted by the Deakin University Ethics Committees (HEAG-H 70-2020). After providing consent, participants completed semi-structured interviews, ranging from 20 to 146 min (*M* = 67 min). The interview schedule was developed by the research team, drawing on relevant literature and the study’s research questions, and was subsequently refined in collaboration with the research study advisory group, which included clinical psychologists, organisational psychologists, and leadership representatives from healthcare organisations. The interview schedule consisted of two sections; the first focused on family functioning and the second focused on workplace experiences. This study reports on the responses in the second section, focusing on workplace supports and challenges experienced by these workers in their work-facing roles. When responding, participants were asked to reflect on changes “since the beginning of the COVID-19 pandemic”.

For context during the data collection period, Victoria was coming out of a lockdown that ended in October 2020. With a length of 112 days, this was the longest continuous lockdown internationally and was rated at almost the highest level of severity on an international stringency index [40]. During this lockdown, regulations provided for four permissible reasons to leave home (work or study; exercise, limited to 1 h a day; shopping for supplies; and medical care and caregiving). State-wide restrictions included the closure of schools, restrictions on household visitors, and limits to movement.

Interviews were completed 1-on-1 over Zoom video conferencing by authors EC, BL, and a research assistant, none of whom had any prior relationship with the participants. Participants were only informed about the study objectives and methodology, as written in the plain language statement. Written and verbal consent were gathered prior to interviews, which were audio recorded. Interviews were then transcribed and sent to participants for review and feedback before being de-identified for analysis. No interviews were repeated. The interview team met regularly and discussed emerging themes and data saturation.

### 2.4. Data Analysis Procedure

This study applied a reflexive thematic analysis [41]. This allowed the team to conceptualise the themes in both explicit and conceptual meaning-based patterns. To prevent losing individual perspectives and experiences, the thematic analysis was conducted ideographically. While interview questions were grounded in topics on the healthcare workplace, the analysis was inductive and data-driven.

A random allocation of 4–8 transcripts was assigned to authors EC, BL, AR, NT, and JS. Initial analysis of transcripts included reading and reviewing transcripts as well as identifying quotes that were meaningful to the research questions, participant, and researcher. Authors then coded each transcript at a micro level before clustering them to identify initial concepts. These concepts were then compared and contrasted in a reflective discussion with the team as a whole. In these discussions, preliminary super- and subordinate themes were developed. Each transcript was then reviewed a final time against these preliminary themes for consistency and contrary cases. Super- and sub-ordinate themes were finalised in a second team discussion. To preserve authenticity and individual perspectives in the results narrative, the lead author referred to original transcripts as well as a tabulated resource of all participant codes and themes. The analysis team also examined participants’ demographic information (e.g., managerial status, gender, years of experience) to assess whether different role groups produced distinct thematic patterns. The emergent themes were then reviewed for subgroup differences, and any demographic-related variations that were identified were reported in the results.

### 2.5. Reflexivity

Data collection and analysis were led by EC (female) and JS (female), while the manuscript preparation was led by both authors as well as BL (male). At the time of writing, EC was a Psychologist and Research Fellow, while BL was a PhD candidate and a research fellow, and JS was a Clinical Psychologist and Professor. Both EC and JS were working with families and parents as psychologists and researchers while also being parents in Victoria, Australia, during this period. The broader authorship and research team brought experience from their work as frontline HCWs and management in hospital settings and expertise in qualitative research. The team thus had, and was shaped by, a shared paradigm of these experiences, which impacted their viewpoints. To maintain data integrity and procedural rigour during data collection and analysis, the team engaged in journaling during the interview and analysis stages, self-reflection, and team discussion regarding emerging issues and findings.

## 3. Results

Three overarching and five subordinate themes were identified in the data and are summarised in Table 2. Quotes are italicised, with each participant’s demographics bracketed after quotes (gender, age, profession).

### 3.1. A Rapidly Changing Workplace

Within this theme, participants reflected on the rapid changes in information, procedures, and protocols surrounding infection control. For many, these rapid changes brought with them a new level of fatigue and uncertainty in the workplace. Within the JD-R framework, the dynamics described in this theme functioned predominantly as work-domain demands, characterised by their volume, velocity, and concurrence rather than by any single dominant stressor. Together, these stressors placed a significant physical and mental burden on them, which inadvertently affected family life.

### 3.2. Uncertainty in the Workplace

Participants noted that the changes at work were rapid, and the implementation of new protocols was often immediate and required additional work demands, particularly for those with leadership responsibilities. The sustained uncertainty experienced by frontline HCWs operated as a cognitive demand, depleting attentional and decisional resources over time.

One nurse manager noted that implementation was usually *“now or on Saturday mornings”* and they had to work *“on weekends to actually make sure that [staff] were okay”* (M, 38, Nurse Manager). As a consequence, participants noted there was little time to adapt to these changes, with one citing it was *“a real shock to [staff] initially that [they] were going to be in this COVID environment”* and *“didn’t have much time to get used to [it]”* (F, 46, Allied health), and another cited *“it was a difficult time to work obviously. Yeah. Just with—yeah—everything changing so quickly, so rapidly… it’s crazy”* (F, 35, Assistant Nurse Manager).

With such rapid changes and risks at work, participants described increasing uncertainty in their workplace, creating a sense of being out of control, and increasing their worries. One participant had “*a real fixation on when is this going to end; when is this going to end; when can I go back to normal?”* (F, 33, Allied health), while another noted there was an *“unknown of what the workplace was going to look like and the potential for that being catastrophic”,* which was *“quite scary as well”* (F, 31, Nurse). Leaders themselves were concerned and had increasing pressure and uncertainty with managing risk and infections among their staff. One cited being worried about “*what I’m going to need to do, should we have a positive staff member that’s been working for the last 3 days in the department… How many staff are going to have to go off on furlough? … What this is going to look like to the media? …Are they going to actually blame us”* (M, 38, Nurse Manager). There was also a level of uncertainty and worry associated with the impacts of their work on family life and family members, such as being forced to isolate from them. For example: *“If I had to be isolated in a hospital setting that the government has set up now for health care workers, if they are impacted, they have to be isolated from their own families. What impact this is going to have on my wife, so that affects my mum and dad, and shopping and everything”* (M, 44, Allied Health).

### 3.3. The Impacts of Rapid Change

For many, these rapid workplace changes resulted in a level of emotional and cognitive burden on staff. For some, this started from a sense of information overload. One participant said, “*it just felt like the goalpost[s] kept on changing with the more information that obviously came out. And that I think in itself was overwhelming to begin with”* (F, 31, Nurse). For others, it was the planning process itself that was overwhelming. Participant (F, 39, Nurse) noted: *“The mental preparation for it and then the physical preparation… protocols were changing constantly… The physical ICU was changing… so it just felt like we were preparing for war.”*

The procedural changes with wearing full PPE and its actual experience were also fatiguing, anxiety-provoking, and an additional burden. Some comments from participants were:

*“You’re already drained from being in full PPE, and you can’t drink when you want to. You can’t eat when you want to… So there’s an extra mental stress on top of you that just depletes you.”*

(F, 33, Nurse)

*“It was really scary at the start, being immunosuppressed and being told not to wear a mask … so that we can conserve them for later when it will be a high risk.”*

(F, 41, Allied Health)

These uncertainties also led to a tremendous strain on mental wellbeing, with participants illustrating boundary-spanning impacts. Work-domain stressors crossed into the family domain and back, indicating that the demand–resource balance for these frontline HCWs operated across rather than within a single domain.

One participant expressed, *“there was so much mental strain because every single day, you’d go to work, and something had changed… It was so mentally draining to have to try and keep up”* (F, 42, Nurse). This then led to an additional strain on family relationships when participants were drained at work and brought home their stress. For example: one participant commented that the there was a “*whole new world of work, and it’s even more draining. So it just had a huge impact on my relationship with my youngest son”* (M, 44, Allied Health) and another felt that *“at the beginning when this all started there was a lot of angst and a lot of stress and certainly didn’t feel that I was as present when I got home.”* (F, 29, Nurse).

### 3.4. Workplace Support

In this theme, participants described their experience of supportive resources within the workplace, with specific references to organisational, local management, and collegial supports in response to the workplace impacts during the COVID-19 pandemic. Specifically, participants noted times when these supportive resources were lacking and when they were helpful, as well as their implications for family life.

#### 3.4.1. Organisational Supports

Organisational support operated as a multi-dimensional structural resource spanning physical provisions (PPE and safety procedures), informational mechanisms (transparent communication), formal wellbeing programs (EAP), and indirect family-safety consideration; the resource was protective when responsive to evolving demand and contextually targeted but eroded confidence and trust when supplies lagged behind risk or when formal supports were generic and “fell flat”.

Regarding organisational support around infection control, PPE supplies were initially limited and restricted, which led to some feeling like their safety was disregarded by their organisation. When describing this experience, participant (F, 46, Physician) said: *“It’s like sending soldiers out over the frontline without helmets or without bulletproof vests when they’re available,”* and participant (F, 42, Nurse) said: *“that little bit extra fear and that little bit extra uncertainty and annoyance. I don’t feel like, as an organisation, my workplace really addressed that at all.”* However, participants acknowledged that organisations were also impacted by the pandemic and were trying to adapt. One participant said: *“I don’t think the organisation did as much as they possibly could, but I don’t think they really had any—you know, there were—they were only just landing on their feet trying to figure everything out.”* (F, 29, Nurse)

Nevertheless, most participants reported that access to PPE and its associated procedures quickly improved, which was important to help staff feel safe, assured, and supported by their organisations. When reflecting on this experience, one participant noted: *“I’ve actually been really impressed with how [Organisation] has looked after me personally with being pregnant. And sort of the wider ICU unit has really risen to the occasion, and I’ve continued to feel really safe”* (F, 31 Nurse). Increasing workplace confidence and feelings of safety were particularly apparent when communication of critical information improved and was more transparent. For example, one participant expressed that “*As the pandemic went on, they got much better at sharing what was happening”* and *“If we had corona cases, we knew about it. They definitely probably made mistakes, but they rectified them and stepped up to the challenge each time to make the processes better and things like that.”* (F,40, Nurse Manager). Transparent communication was especially helpful in subduing their worries that arose from the media’s aggressive representation of COVID-19. One participant noted that when there was transparent communication, *“[It] wasn’t as bad as what the media was sort of showing anyway. So it was almost a bit of a relief”* (F, 31, Nurse)

Regarding work risks on family safety, some staff felt it was not considered initially, but were later considered indirectly through safety planning for staff. One participated quoted: *“So yeah I don’t think their first thought was for how it would impact the families of the staff but that did definitely then become an issue and was definitely you know taken into consideration”* (F, 56, Nurse Manager) and another noted: “I can’t say that my workplace went out of their way to do anything from a family’s perspective. I think it was …keep their staff safe…that would keep families safe.” (F, 46, Dietician). Additionally, organisational support, in the form of practical support, was appreciated in some workplaces where they were implemented. *“They’ve got a concierge service that they have people, so you know, if there’s a worker that can’t go shopping or can’t do things, then they’ve actually got people to do that.”* (M, 46, Nurse Manager)

In terms of formal wellbeing supports, such as employment assistance programs (EAP) and wellbeing interventions, some were reassured through their use, and it helped address their concerns, *“I accessed it a couple of times just to make sure my head was in the right space for my team, my priorities were focused and that probably made me a stronger person outside of work as well…I want to keep myself basically safe as well.”* (M, 34, Nurse Manager). However, they were not always well targeted to the needs or cultures of some teams. One participant stated that: “*EAP sessions that they set up for us, generally, they generally fall really flat … it never really seems to hit the mark… If there had have been potentially something at the start that was alleviating the fears of what we were going through… then that may have been better at the start”* (M, 38, Nurse Manager), and another example was: *“They have a program at work where there is a group of people who are trained to be like supportive counselling type people, but I didn’t really know any of those people. I wouldn’t really go to them for help”* (F, 33, Allied Health)

#### 3.4.2. Local Management

Local management emerged as a high-leverage social resource for frontline HCWs but one carrying a structural cost: the same leaders who absorbed organisational stress on behalf of their teams became a population of frontline HCWs whose own demand–resource balance was systematically inverted.

For staff with management and leadership responsibilities, many held unofficial dual roles, which were exhausting for many as they needed to support their teams while also managing their own personal distress and concerns. Some comments from participants included:

*“All I could do was reassure them that we’re doing everything in our power to make sure that we’re protected and that we’re not going to end up like those places that you see on the news. But that’s exhausting… bearing the anxiety of… 180 nurses, 30 clerical staff, 10 nurse practitioners, a handful of care coordinators, and then the other 100-odd doctors.”*

(M, 38, Nurse Manager)

*“I’ve been a nurse manager for many years, but I’ve never had to… deal with a health crisis that I could take home and my staff could take home and give to their families.”*

(F, 56, Nurse Manager)

In terms of staff’s experiences with workplace measures and support from local management, such as direct supervisors and line managers, flexibility with leave was noted as important to participants, as it assisted with managing family and additional caretaking responsibilities that came with the lockdown restrictions. *“My manager was supportive of me taking a day off a week…he was aware that it was because I had home schooling and things to deal with…They did have that consideration for family”* (F, 46, Dietician). It was also helpful to participants when they experienced a sense of openness in the workplace to acknowledge challenges and seek assistance. *“Openness and permission about saying that if somebody felt sick during a shift, that they—they acknowledge it and … report it, rather than work through the shift…If they felt uncomfortable doing something,… there’s some way to work around that—if someone felt very strongly about it.”* (F,33, Physician). Some participants also noted that management recognised the burden on staff and enabled non-essential work to be reduced or stopped while planning for the response, which was helpful with prioritising and managing workloads. An example quote was: *“They …did recognise that we can’t do the standard daily work right now…we stopped a lot of auditing for a small period of time… non-essential work was excused so we focused on the here and now and the surge planning”* (M, 34, Nurse Manager)

#### 3.4.3. Peer Supports

Informal peer support also functioned as a valued social resource for frontline HCWs, both a source of practical knowledge-sharing and a buffer against the emotional load of frontline work. Collegiality within teams was noted as an important part of coping strategies for many participants. Between colleagues, participants described an active involvement in supporting each other. An example quote was *“there was definitely lots of support in from the internal group…[and] from each other… “What are you doing? How are you doing this? What do you do with your shoes when you go home?”, all these sorts of things that you discuss.”* (F,49, Allied Health). Collegial support also came in the form of mental health monitoring and assistance. One participant noted this, *“a few colleagues who really were actively having adjustment reactions, borderline suicidal, this pandemic… I found myself doing a couple of rescue visits to them and taking them off work for six months to go and see psychiatrists.”* (F, 46, Physician)

However, this resource proved structurally vulnerable to the same infection-control protocols designed to manage workplace demand, illustrating a recurring dynamic in which demand-management responses inadvertently deplete the resources that buffer subsequent demand. For example, *“The tea room was rearranged…. it wasn’t sort of the warm, friendly space that you could go and sort of chat and support each other, it felt a little bit cold”* (F, 39, Nurse). As a consequence, several participants noted the absence of face-to-face peer debriefing and collegial support, which was difficult because it was part of their usual strategies for managing work demands. Specifically, participants felt that this impacted the support system that understood and related to them the most. One participant (F, 39, Nurse) noted that *“You do feel sometimes like the only people that really understand what it’s like are the people that work with you”* and *“it’s just so important for us as a group of staff to have that connection and to know that you’re all in it together [but] COVID … has made that connection with others quite difficult.*

### 3.5. Work and Career Reflections

The reflections in this theme describe outcomes of the demand–resource imbalance described in earlier themes. Pride and meaning operating as personal resources that sustained engagement for some, while reduced career enjoyment and exit intention emerged as strain outcomes when demands chronically exceeded resources.

A sense of pride was reported by some participants in their personal contribution to the COVID response, helping them cope and stay motivated despite impacts on work in healthcare. One participant noted: *“I was quite proud to be one of those people who were out sort of working and doing something to help…It definitely helped me to cope with what I was doing because it made me feel like it was worthwhile, and it was my contribution to the pandemic.”* (F, 39, Nurse)

Partners and family members also had pride in participants’ frontline roles and were generally very supportive of their work. This also helped participants maintain a level of satisfaction and pride with their frontline work. For example, *“He was on the whole just so supportive, and I think quite proud of what I was doing, and my kids were too … they’d say … thanks for your hard work and they’d see how tired I was when I got home …. bring me drinks and stuff, they were really looking after me ….It was lovely, and it made a really big difference for me.”* (F, 39, Nurse)

There was also a level of satisfaction and hope with the response within their local environment, and that the local experience had been less severe, compared to other countries, both professionally and personally. Participant (M, 46, Nurse Manager) noted that *“I think we’re pretty much the only country that’s—or the only jurisdiction that’s really properly beaten down a decent second wave… It does show that it is possible to control this thing, so there are hopeful signs as well”*.

However, there were also others who felt a reduced enjoyment or pride in their work due to the increasing stigma and demands of being an HCW. In regard to stigma participant (F, 35, Assistant Nurse Manager) said: *“I was sort of hiding the fact that I was an emergency nurse– because you … don’t want people to be like… you’re contaminated… it was sort of a bit of a flip”*, and in regard to demands at work, participant (F, 38, Nurse) said *“I don’t like going to work anymore, not as much …We have COVID, suspected COVID beds that we had to work in quite a lot in the full PPE…Everything’s harder when you’re wearing PPE. And …the extra responsibility.”* Participants were also conscious about the long-term mental health consequences due to the COVID-19 pandemic. An example quote is *“I’m conscious of the mental health impacts of the team, my team, probably myself and my fiancé, my family/parents; it could be quite an impact if mental health does become a problem, but we don’t know that yet.”* (M, 34, Nurse Manager)

### 3.6. Overview of Findings

Across the three themes identified, three cross-cutting patterns emerged: workplace conditions were defined by rapid and concurrent workplace changes rather than any single dominant stressor; the resources participants found most protective were also the most structurally degraded by the pandemic response (leadership cost, peer-support erosion, poorly targeted EAP); and work-domain stressors crossed into the family domain for these frontline HCWs with parenting responsibilities.

## 4. Discussion

Study findings highlight how frontline HCWs’ wellbeing and support systems can be affected under a strained healthcare system. Although these findings emerged from the pandemic, the underlying dynamics extend beyond it and inform present-day workforce challenges. Examined under the Job Demands–Resources lens [23,24], these findings extend the framework in three ways. First, leadership functioned as both a resource and added demand depending on role (a resource-cost transfer up the hierarchy); informal peer support was depleted by the same protocols designed to manage demand, and several demands crossed the work–family boundary, indicating limits of within-domain JD-R applications.

Frontline HCWs during the COVID-19 pandemic in this study have reported that when hospitals face workplace crises, it can trigger rapid organisational changes, increased demand, and heightened uncertainty, which can subsequently place considerable pressure on staff wellbeing and the capacity of workplace support systems.

To support their needs in times of workplace crises, frontline HCWs in this study emphasised the importance of organisational support, specifically through organisational communication that was open, transparent, and responded to the immediate concerns of the healthcare workforce. However, the findings suggest that with increased workplace support, there can also be an additional burden for leaders providing these supports, a mechanism rarely surfaced in literature focused on frontline staff outcomes. Middleton and colleagues [42], in their study of Australian nurse managers during COVID-19, documented elevated psychological burden among managers and noted competing demands from staff and senior management; the present study contributes the JD-R-level account of how that burden is generated, a resource-cost transfer up the hierarchy that is rarely articulated in the broader frontline HCW JD-R literature [43,44].

Peer support was also identified as an important coping mechanism for frontline HCWs but was structurally disrupted by the same infection-control protocols designed to manage workplace demands. This contrasts with pre-pandemic systematic reviews by McKinley et al. [45] and Lim et al. [46] and pandemic-era peer-support evaluations [47,48,49], which have treated peer support as a stable protective factor without examining how changing workplace conditions may erode peer interactions.

While the increasing demands and experiences of stigma from the community have led to growing concerns about the future of healthcare work, the findings suggest that their social and workplace support systems can play a role in positive career satisfaction, highlighting an important focus for workforce retention strategies in modern healthcare systems.

### 4.1. Managing Workplace Changes

The pandemic has shown how healthcare systems function under conditions of crisis and sustained strain, revealing enduring strengths and vulnerabilities in workforce and family support, leadership capacity, and role sustainability that remain relevant beyond the acute crisis period. Consistent with previous research [50,51,52,53], this study found that the COVID-19 pandemic led to health care teams experiencing a complete change in workplace procedures and practices, which has subsequently led to a rise in psychosocial stressors in the healthcare workplace. More specifically, the current study found that the rapid and frequent changes to working procedures and infection control protocols during the pandemic were accompanied by overwhelming work demands, uncertainty at work, and a lack of job control among frontline HCWs, the high-demand, low-control combination identified by Karasek [25] as the primary driver of work-related strain. Consequently, these work-related stressors have led to reduced career enjoyment, increased psychological burden, and familial impacts. While workplace changes are designed to improve working environments and safety, these findings reinforce that they can lead to the emergence of unforeseen stressors that can pose significant risks to mental health and wellbeing [54,55,56]. It is therefore important for organisations to consider how they can support frontline HCWs’ mental health when adapting and implementing changes in their workplace, especially during times of distress and trauma.

### 4.2. Leadership and Supervisor Support

A recommended strategy when navigating workplace changes has been through support from leaders throughout the process [57]. Consistent with prior work [43,44], findings from this study show that positive support from leadership during the pandemic helped frontline HCWs manage stressors and feel motivated at work. The present study extends this evidence by identifying that frontline HCWs value a *multi-faceted approach* from leaders when providing support during times of distress, one that explicitly includes support for family life. This finding connects leadership support directly to the work–family boundary, a connection that is largely absent from leadership-support literature [58,59,60] focused on within-work outcomes (e.g., burnout, stress, turnover).

The findings in this study specifically highlight that positive supports included proactive and pre-emptive steps to ensure the safety of HCWs as well as their families, reconsideration of workload and role responsibilities to account for the changing environment, and providing valued wellbeing supports such as reassuring and transparent messaging, formal supports, and flexible leave and work arrangements to accommodate family life. Frontline HCWs’ descriptions of valued leadership behaviours in the present study provide empirical support for the five normative leadership recommendations proposed in Stoller [61] 2020 commentary on healthcare leadership during the pandemic: (1) proactively anticipating events and creating contingency plans accordingly; (2) clearly clarifying governance for the crisis; (3) implementing action plans that ensure both employee safety and their wellbeing; (4) providing frequent and iterative communication, and (5) offering realistic, optimistic and transparent messaging to employees that acknowledges difficulties and offers hope. Where Stoller [61] derived these recommendations from professional reflection, the present study contributes the frontline HCWs’ own accounts of which of these leadership behaviours were valued in practice during a sustained crisis, distinguishing those that frontline HCWs experienced as genuinely supportive from those that operated only as managerial intent.

Many agree that leadership plays an important role in frontline HCWs’ workplace wellbeing, and is a critical priority in organisational mental health strategies [58,59,60]. But it is important to recognise and consider the burden this can place on leaders themselves. Those in managerial and supervisory positions in this study reported that the increased need for multi-faceted support from staff led to significant pressure for them as they took on additional work while also stepping into unofficial support roles that left them exhausted and worried for their staff. These findings further support evidence that shows healthcare leaders can experience stress and psychological burden associated with their leadership roles, in addition to what their staff experienced during the pandemic [42]. The present study highlights the mechanism-level detail of this leadership-burden literature by identifying how the cost is generated, through absorbing unofficial support roles for staff during demand spikes, often without compensating resources or organisational support.

This is concerning given that leaders play a central role in the healthcare system in supporting frontline HCWs’ mental health and wellbeing. Given this, there is a need to also include strategies to support leaders with the additional burden they may carry.

### 4.3. Peer Support

One approach to consider is to preserve and enhance peer support in the healthcare workplace. McKinley et al. [45] systematic review of resilience in medical doctors, conducted prior to the pandemic, identified peer support as a consistent protective factor across studies. Findings in the present study extend this work, suggesting that peer support is highly valued and widely utilised by frontline HCWs to cope with challenges and demands at work during the pandemic. It has also been evidenced that peer support is associated with an increase in resilience and coping, and can significantly protect against the mental health challenges during the COVID-19 pandemic [47,48,49]. However, frontline HCWs in this study identified that social distancing restrictions at work had a substantial impact on their ability to provide and receive support from their peers. The present findings demonstrate that the infrastructure enabling peer interaction is itself contingent on workplace conditions and can be structurally degraded by infection-control responses. Within the JD-R framework [23,24], this represents a previously under-described dynamic in which a demand-management response (here, infection-control protocols) inadvertently degrades a job resource (peer support), shifting the resource-provision burden onto another resource (leadership) and compounding the leadership-burden dynamic discussed above.

Given these findings, it appears that prioritising peer support during times of distress can have a two-fold impact. By enhancing and preserving peer support between staff, organisations can inadvertently help divert the reliance on leaders for support and, in turn, elevate some of the burden on them. Several formal peer support programs implemented in healthcare workplaces during the pandemic have now shown promise and offer some guidance on their development [62,63,64]. Peer support programs can provide frontline HCWs with a support network they can effectively rely on to quickly understand their experiences, help identify their needs, direct them to relevant resources, and reduce help-seeking stigma.

## 5. Limitations and Future Directions

This study interviewed 39 frontline HCWs with parenting responsibilities recruited primarily via one major hospital network and specific wards where COVID patients were managed. While we made every effort to recruit a diverse sample, the nature of this study, including the longitudinal commitment, discussion of personal experiences, and length of interviews, may have been barriers to participation for some groups, and thus, it is not certain that all unique experiences have been captured. Additionally, the data collection duration may also act as a limitation in the study. This study interviewed Australian frontline HCWs with parenting and family responsibilities between October 2020 and February 2021. While there are advantages to this sampling frame (i.e., this timeframe covered the two most significant periods of lockdown in Victoria, where many of the participants were located), the rapidly fluctuating nature of the pandemic means that there would be some further diversity in the experiences reflected in the interviews. Several additional considerations apply to interpretation. Recruitment from a single metropolitan health-service network constrains transferability to rural/regional, smaller, or non-public-sector contexts; self-selection of more-affected or more-available frontline HCWs cannot be ruled out; the parenting-responsibility eligibility criterion means findings should not be generalised to frontline HCWs without dependent children.

Three directions for future research follow from these findings: longitudinal follow-up of frontline HCW parent participants into the post-acute pandemic period to clarify which dynamics persisted or compounded; comparative work across rural/regional and non-public-sector contexts to test transferability of leadership-cost and peer-support-erosion findings; and intervention-development research on work–family-aware organisational policy, leadership-capacity programmes, and protected peer-support infrastructure under altered protocols. Parallel work with frontline HCWs without parenting responsibilities would also help disentangle which dynamics are specific to the parent-frontline HCW intersection.

## 6. Conclusions

Overall, the findings of this study have shown the unique challenges faced by frontline HCWs during the COVID-19 pandemic, which placed a significant psychological burden on the workforce. Although data were collected during the pandemic, the study identified broader system-level dynamics and support needs that emerge during periods of sustained strain, conditions that continue to contribute to workforce shortages and retention challenges in healthcare systems today. The dynamics described (i.e., demand–resource imbalance, leadership-as-burden, and peer-support erosion under formal protocols) can recur under any sustained healthcare-system stressor (workforce shortages, climate-driven surge events, conflict-related workforce/supply disruptions), making frontline HCWs’ COVID-19 experience a guide for future responses rather than a historical record. Embedding leadership development, protecting peer-interaction space, and pre-positioning work–family-aware policy translate this learning to current workforce sustainability efforts. Given that healthcare systems globally are currently facing a workforce crisis, the findings of this study offer important insights to inform contemporary workforce policy and practice.

## Figures and Tables

**Table 1:** Participant demographics.

	n	% of Parents
Gender		
Female	33	84.6%
Male	6	15.4%
Age range		
Under 30	1	2.6%
30–34	6	15.4%
35–39	8	20.5%
40–44	12	30.8%
45–49	8	20.5%
50–54	1	2.6%
55–59	3	7.7%
Developmental/school stage of children *		
Pregnant	4	10.3%
Preschool	15	38.5%
Primary	22	56.4%
Secondary	13	33.3%
Higher and vocational education	2	5.1%
Relationship status		
Separated/divorced	3	7.7%
Married/de facto	36	92.3%
Cultural Background		
Asian	5	12.8%
Australian—non Aboriginal or Torres Strait Islander	31	79.5%
British or European	2	5.1%
Other	1	2.6%
Education		
Diploma	2	5.1%
Graduate degree	12	30.8%
Post-graduate degree	25	64.1%
Location		
Rural/Regional/Remote	6	15.4%
Urban	33	84.6%
Profession		
Allied health (e.g., Psychologist, Pharmacist)	14	35.9%
Nursing	21	53.8%
Physician/Medical practitioner	4	10.3%
Held managerial duties		
Yes	9	23.1%
No	30	76.9%
Department		
COVID Ward	5	12.8%
Emergency Department	24	61.5%
Hospital in the Home	5	12.8%
Intensive Care Unit	5	12.8%
If partnered, was partner actively working away from home		
No	19	48.7%
Yes	20	51.3%
Perceived that they are at risk of Infection		
No	27	69.2%
Yes	12	30.8%
Perceived that a family member at risk of Infection		
No	25	64.1%
Yes	14	35.9%

* Totals greater than 100% as several parents had children in more than one age grouping.

**Table 2:** Themes and subthemes identified.

Theme	Sub-Themes
A rapidly changing workplace	Uncertainty in the workplace The impacts of rapid change
Workplace supports: The Status Quo	Organisational supports Local management Peer supports
Work and career reflections	

## Data Availability

The datasets used and/or analysed during the current study are available from the corresponding author on reasonable request. The data are not publicly available due to privacy and ethical restrictions.

## Supplementary Materials

The following supporting information can be downloaded at: https://www.mdpi.com/article/10.3390/healthcare14101400/s1.
