# Supplementary material for: “The Only People That Really Understand”: A Qualitative Study of Healthcare Workers’ COVID-19 Experiences and Implications for Workplace Support
Source: Healthcare (Basel). 2026 May 20;14(10):1400. doi: 10.3390/healthcare14101400 (PMC13205624; doi:10.3390/healthcare14101400)
Supplement: Supplementary file 1 [file healthcare-14-01400-s001.zip › Interview schedule.pdf]

COVID-19 and FHWs: Adult Interview Questions

Date: *April 2020*

Full Project Title: *Investigating the impact of the COVID-19 pandemic on Victorian healthcare workers and their families.*

SECTION 1: PREAMBLE

Thank you for agreeing to participate in this interview.

The aim of this interview is to gain an understanding of the impact of the COVID-19 pandemic on the way your family is functioning. If you are a healthcare worker, you will also be asked specific questions regarding the impact of your work activities later in the interview.

I just need to check that you have received and read the information statement that I sent you?

I also need to let you know that your participation in this research is entirely your choice. Also, it is entirely up to you whether you want to answer all of my questions. So, if there's a question that you'd rather not answer, you can just let me know and we'll move straight on to the next question

Finally, all the data that I collect from you will be treated in a confidential manner, so I'll be removing all information that identifies you from the transcript, and you can check that yourself because you'll be receiving a copy of the transcript before I do any analysis so you can make any desired changes to it.

I also want to remind you that these interviews are confidential, what you say today won't be shared with other family members (or your workplace if you are a health care worker) and we will not include any personal information in our results. The only exception to this will be if we have any concerns about the safety of yourself or someone else. With that in mind, we would also ask that your answers to these questions are focused on the family dynamics rather than personal details about individual family members.

Any questions so far?

Even though I understand that you have provided consent to having this interview being taped, I would like to ask you again, whether you are okay with having the interview recorded? (If yes, start recording)

SECTION 2: Healthcare worker specific questions

- Has your partner raised any specific concerns regarding your role as a healthcare worker through this pandemic?
- Have your children raised any specific concerns regarding your role as a healthcare worker through this pandemic?
  > what has been the impact of this on you, your partner and your children?
- If yes, how have you addressed these concerns as a family?
- Possible follow up: how has your prep/return from work routine changed during the pandemic?

- What concerns, if any, do *you* have regarding your role as a frontline healthcare worker during this pandemic? If so, what are these concerns?
- What concerns for your family, if any, do *you* have regarding your role as a frontline healthcare worker during this pandemic? If so, who are you concerned about – your partner, kids? And in what way?

> Have you communicated these with your family?

> How have you managed these?

- What strategies, if any, have you put in place to manage the additional demands associated with your work at present?
- ➢ Probe: supervision, debriefing, support, self-care, boundaries etc?
- ➢ Follow up: Do you have any control/autonomy over your management strategies?

- What strategies, if any, has your workplace put in place to manage the additional demands associated with your work at present?
- Do you feel that your workplace has considered families in their decisions since COVID?

SECTION 4: CONCLUSION

That is all the questions I have for you. Are there any other comments, issues or concerns you would like to make about this?

I will be sending you a transcript of this interview for you to check and delete any information you think might be potentially identifiable or to add anything you might like to add. You will have two weeks to do this but if you need more time please let me know. What is the best way for me to get this to you?

Thank you so much for your time. We appreciate it. I need to remind you that if you find this process distressing either now or later there are people to talk to. Just let me know and I can arrange this for you.
