# Supplementary material for: “The Only People That Really Understand”: A Qualitative Study of Healthcare Workers’ COVID-19 Experiences and Implications for Workplace Support
Source: Healthcare (Basel). 2026 May 20;14(10):1400. doi: 10.3390/healthcare14101400 (PMC13205624; doi:10.3390/healthcare14101400)
Supplement: Supplementary file 1 [file healthcare-14-01400-s001.zip › Theme Development Table.pdf]

ID	Workplace support	Work routines/ procedures	OTHER
1*	Working long hours (p14) but no sense of annoyance with it<br><br>Early access to PPE a problem but organisation communicated and did what they could about it (p16)<br><br>Positive work supports and examples of maternity and social work giving self-care suggestions (innovative) (p18)	Change in work responsibilities (p13)<br><br>Innovative solutions to problems (p14 and 16)<br><br>Interesting but challenging time professionally (p14)	
5	Major change in what work looks like (compared to other industries, where it was change in comms) p28<br><br>Feeling that organisational response much better in 1st than second wave – more ingenuity, flexibility p30-31<br><br>Doing pulse surveys etc, and offering assistance 6 months in is too late – patterns established p32-33<br><br>Some attempts for support fell on deaf ears, not well suited p 33<br><br>Support at the start could be better, but with so much going on, don't know how what else they could have done – p35	Burden of workload, harder to have boundaries when wfh (p1)<br><br>Increased work demands on already full load p4-5<br><br>Lots of change, implementation decided late on Friday meant needing to work over weekend and concern re information overload for staff p6-7, p18-19<br><br>Changing requirements re guidance and feeling overwhelm p 21	Context – NUM, 2.5 toddler, wife also nurse, pregnant with #2<br>Able to take extended parental leave when next child born Jan 21<br><br>Poor sleep patterns, PlayStation till midnight to unwind, heavy drinking, but noticed this and took action, p 11-12<br>Hard to manage stress, wind down without alcohol p19<br><br>Lack of other conversations – lots of focus on COVID, what is happening etc, No cut off or boundaries p12<br>Although had plans in place for managing quarantine, when it came about, it was v quick p26<br><br>Personal coping strategies – sees this as professional triager (ED role) p29
6	Optimism as a source of protection (p2)<br><br>Getting tested was reassuring (p3)<br><br>Additional workload (p4)<br>Caring role for staff under their supervision (p6)<br><br>Making sure his staff were empowered (p7)<br><br>Impact of schools closing on some staff and trying to support them in that (p7)<br><br>"it's a career changing event really" (p7)<br><br>Support for staff being open and transparent (p7)	More regular contact with staff (daily briefings) (p7)<br><br>Constant change at work (p8)<br>Working overtime to address it and keep staff safe (p8)<br><br>Non essential work excused for surge planning (p8)	
7a	Talks to husband bc he is nurse and understands – rather than seek support from work p. 9<br><br>Workplace considers family life as secondary p. 14 but is important p. 14	Not applicable	Religion a support for this family<br><br>Technology concerns re daughter
EC 7b	Don't know p. 11 (a male)	Not applicable	
8	Felt well informed at work (p.13) Information from DHHS via director was helpful and reassuring (p.10,11)	Adjustment to work schedules to manage childcare (p.4, 5)	Decisions around different childcare arrangements between lockdown 1 & 2 (p.4)

ID	Workplace support	Work routines/ procedures	OTHER
1*	Working long hours (p14) but no sense of annoyance with it<br><br>Early access to PPE a problem but organisation communicated and did what they could about it (p16)<br><br>Positive work supports and examples of maternity and social work giving self-care suggestions (innovative) (p18)	Change in work responsibilities (p13)<br><br>Innovative solutions to problems (p14 and 16)<br><br>Interesting but challenging time professionally (p14)	
	Going to work and putting things in perspective compared to media reports and rest of the world was helpful (p.10)<br><br>Felt supported by workplace and safe re pregnancy (p.13)<br><br>Participant's specific team were supportive, flexible and considered families (p.14)		Conversation with partner to manage challenges (p.5)<br><br>Daughter adapting to others wearing masks (p.6)<br><br>Family going to participant for the answers and holding a sense of not having the answers, being in a situation that was new and unknown (p.11)<br><br>Differing boundaries between participant and other family members (p.11)<br><br>I
10	Workplace support was good towards participant as a manager. Felt the practical support was important and useful. P.9/10<br><br>Adequacy of PPE was important as there were many other factors to worry about. Such as adapting a new role. P.10<br><br>Lack of psychological support in the beginning. P.18	Sudden change in the ward's roles was difficult. Supporting staff during these changes were stressful and exhausting. Lots of concern and reassurance for staff. P.8,15<br><br>Substantial Increased amount of mortality P.8<br><br>As a manager during this time, concern and worry about staff infection was at an individual level. This was stressful. P.9<br><br>As a manager they had multiple roles. Had to provide psychological first aid in addition to other work. P.10<br><br>Participant was emotionally exhausted. P.10	Concern about the impact on Children's social life p.7<br><br>Felt responsibility to the team participant manages and took a shorter leave than she wanted. P.11<br><br>Being strict on staff was not easy. P.13<br><br>Participant as a manager was protective of staff. provided flexibility in wearing more PPE if staff wanted to feel more safe. P.14
12	While work is stressful morale is up – as colleagues have all come together - p. 13<br><br>Workplace is safe so safer for families p. 13	Not applicable	Very much resigned and accepting of situation and "gave up" on discipline and on schooling
15**	Work was supportive of family before the pandemic and continued to be p. 24<br><br>The staff look out for each other bc "all have families) (collegial support) p. 24	Not applicable	Communication with kids about lockdown and virus was a big and useful thing to do
16	Workplace tight around info early on, found out some info from news first, Sharing information, being transparent helped feel safer p13-14		Impact on developmental n3eeds, socialisation for toddler, 9yo with ADHD p7

ID	Workplace support	Work routines/ procedures	OTHER
1*	Working long hours (p14) but no sense of annoyance with it<br><br>Early access to PPE a problem but organisation communicated and did what they could about it (p16)<br><br>Positive work supports and examples of maternity and social work giving self-care suggestions (innovative) (p18)	Change in work responsibilities (p13)<br><br>Innovative solutions to problems (p14 and 16)<br><br>Interesting but challenging time professionally (p14)	
	Mngt actively listening to frontline staff, and responding to issues – not perfect, but rectified and kept trying to improve.p14<br><br>Workplace support, spotters etc, increases level of comfort/safety, Local peer and mngt support, comforting to know there are other supports if needed p22		More worried about psychosocial wellbeing than contagion, esp when not parenting as she would like to p14<br><br>Change in ability to self-identify distress, need to self-sooth p18
17	Husband wfh meant he was home with kids (previously had to work around his schedule p2).<br>FHW was going out, doing all shopping etc and felt it was harder on him (93).<br>No longer getting support from her dad, increased household cleaning needs, but family didn't really pick up on this, even though her hours increased.<br>Has developed more appreciation for support from dad, but also being more relaxed about cleanning standards p4<br>Realising will be OK without level of support (p4)<br>Increased structure around activity etc, use of screens to help provide some guidance p6<br>Balancing teaching and parenting role – in LD2, had to back off schooling more, let things slide, to maintain relationship p7		Context, 11 & 9yo kid
18	Change in workplace culture of feeling sick and working through it to a 'permission' to say something (p.18)<br><br>The importance of open communication from the workplace, not just hearing about things in the media (p.18)<br><br>Support from work "they didn't always get it right, but they tried" (p.19)	Changes to routines and roles at work to reduce risk of exposure to COVID while pregnant (p.16)	More open communication with daughter to explain COVID related changes (p.12)<br><br>Worries about preparing for the 'new normal' (p.15)
19**	PPE lessens anxiety (p17)<br><br>Debriefing and checking their mental health (p17)<br><br>Confidence in organisation to address concerns (p18)<br><br>Checking wellbeing (p19)	We are all working together (p19)<br><br>Flexible work roles (p19)	
24	Adequate PPE (p.13,14)		Homeschooling and managing other children were difficult- felt guilty about neglecting others. (p. 3,4)

ID	Workplace support	Work routines/ procedures	OTHER
1*	Working long hours (p14) but no sense of annoyance with it<br><br>Early access to PPE a problem but organisation communicated and did what they could about it (p16)<br><br>Positive work supports and examples of maternity and social work giving self-care suggestions (innovative) (p18)	Change in work responsibilities (p13)<br><br>Innovative solutions to problems (p14 and 16)<br><br>Interesting but challenging time professionally (p14)	
			Changing routines & lack of activities impacted children's sleep (p.1,2,5)<br><br>Nursing Home unsafe-poorly run, worried about resident's mental health<br><br>Coping strategies- Good family communication and maintaining routines(p.10, 18)<br><br>Increased screen time- to manage children at home (p.2,3,4)<br><br>Worries and struggles with adjusting back (p.2,5,7,8)<br><br>Stigma as a frontline HCW (p.12,13)
26	Role as a HCW helped family adapt – transfer of skills from hospital to home life (p.5)<br><br>Support from family helped her as a HCW (p.16)<br><br>Supports provided by workplace (p.17, 18). NUMS and ANUMs particularly supportive (p.19)<br>Supports needed in workplace (p.17, 18)	Work stress impacting parenting and engagement with family (p.7)<br><br>Change in routines to manage risk of infection and also emotional wellbeing (p.11)	Observed lack of motivation in kids and sense of loss (p.4)
28	Routine very similar but felt more mundane (p. 2)<br><br>COVID was less impactful than husband study demands (p. 2-3)<br><br>Little change in parenting roles – husband previously very busy. Some extra time but busy studying so stayed as mother being primary carer. (p4).<br><br>Outside supports for cooking, Au pair/nanny. Stress of not having access to support at times during lockdown. (p4, 5)<br><br>Children reliant on parents to be their entire world (p7)	Took child out of childcare due to risk of infection (p 1)<br><br>Considered stopping work due to COVID risk (p7)<br><br>Risk greater for her than husband in another medical section (p7)<br><br>Breastfed for longer bc/ of immune benefits for infant (p7)<br><br>Concerns over PPE and risks for family/infant (p7)<br><br>Decision making about childcare (p8)	Brought stress home from work(p4)<br><br>Increased PPE made it harder to keep hydrated and caused health problems (p13, 14)<br><br>Changes and communication (p14)

ID	Workplace support	Work routines/ procedures	OTHER
1*	Working long hours (p14) but no sense of annoyance with it<br><br>Early access to PPE a problem but organisation communicated and did what they could about it (p16)<br><br>Positive work supports and examples of maternity and social work giving self-care suggestions (innovative) (p18)	Change in work responsibilities (p13)<br><br>Innovative solutions to problems (p14 and 16)<br><br>Interesting but challenging time professionally (p14)	
		Friends and extended family not abiding by rules (p9)<br><br>Husband viewed as low risk because of precautions and low incidence (p 12)<br><br>Concern re bringing COVID home to kids – esp early days (p12)<br><br>Concern with colds of bringing COVID to work – calling in sick and getting tested. (p12)<br><br>Impact of work/infection risk on breastfeeding (p12)<br><br>Seeking advice of friends who are infection specialists about work decisions (p12)<br><br>Put off getting pregnant again due to work in high risk environment (p14)	
30	ICU –<br>Wave 1 – organisation scrambling to establish responses etc… Initially allowed to have direct contact, PPE, but no other change<br><br>Wave 2 – weren't allowed in, but would still have to accept referral and find other ways to communicate, e.g. zoom, phone call….Dept split into red/green zones, with SCOVID in red zone,<br><br>Change in ED presentations (more DV, MH), but no major change in coping strategies – just normal prof skills, BUT couldn't work across sites, pick up extra shifts…<br><br>Change in work protocols to ensure protection, but missed out on peer supervision, debriefing due to being in open shared areas….made her more frustrated at home…<br><br>Family support – they tried, manager flexibility was offered for school stuff, and suggestion to be able to alter shifts, but that never really came through. Feel supported by local manager, but not at organisational level.		Developmental nature of challenges, esp key transition (prep) p 3<br><br>Impact on own MH trying to hold it together at home and work with extended family, esp as single parent (p4/5)<br><br>Impact on children – heightened emotions, lashing out, sleep etc p10<br><br>Additional / cumulative stressors (moving house, property settlement re divorce) p 12<br><br>Psych support helping, hard to get perspective when in your bubble (even as health professional) p 13<br><br>Physical space challenges with active kids in small house p 18<br><br>Additional burden impact on MH of parent and children – clingy vs wanting space, disrupted sleep and handovers were harder p 20<br><br>Intense debate about how COVID should be managed within family, led to tension. FHW – not overly worried because was

ID	Workplace support	Work routines/ procedures	OTHER
1*	Working long hours (p14) but no sense of annoyance with it<br><br>Early access to PPE a problem but organisation communicated and did what they could about it (p16)<br><br>Positive work supports and examples of maternity and social work giving self-care suggestions (innovative) (p18)	Change in work responsibilities (p13)<br><br>Innovative solutions to problems (p14 and 16)<br><br>Interesting but challenging time professionally (p14)	
			involved…Issues within family re willingness to risk exposure – parents, initially more cautious, but became more lax in Lockdown #2 to help out. Sister was more concerned…Not discussed overtly
33			Prior open communication facilitated communication about covid during outbreak. P8<br><br>Communication/"early escalation" an important factor in support during work. P 14<br><br>Prior experiences in the ED and with burnout has made participant more resilient and have strategies now. P 14<br><br>Partner's resilience and stability help kept participant's anxieties at bay. P. 11<br><br>Differences between organisational responses/systems. More guidelines and control from organisation was seen as beneficial as it provided more security. P. 14
34	partner supported participant in role as a HCW (p.9)<br><br>Identified need for more support at work (p.12)…emotional impact (p.12)…more transparency and open communication needed (p.13)<br><br>Workplace were flexible however in meeting family needs (p.12)	Returning to work in a time of change (p.2)…worry about procedures in place (p.3)<br><br>Change to routines and impact on family life (p.10)<br><br>Negative impact on communication at work (p.11, 12)	Increased confidence and passion for role as a nurse and new career opportunities (p.13, 14)
39	Support from colleagues<br>P.15<br><br>Responsibility as a team leader supporting staff was challenging P.15	Lack of staff and equipment P 9,10,16	Concerns and caring for parents P 2,6, 9<br><br>Difficulties managing children's screen time<br>P 6,7<br><br>Intentionally allowing screen time due to circumstances P 6<br><br>Using online outlets helped P.15,16<br><br>Adapting lifestyle to maintain routine P 3, 4<br><br>Planning to isolate P 8. 14

ID	Workplace support	Work routines/ procedures	OTHER
1*	Working long hours (p14) but no sense of annoyance with it<br><br>Early access to PPE a problem but organisation communicated and did what they could about it (p16)<br><br>Positive work supports and examples of maternity and social work giving self-care suggestions (innovative) (p18)	Change in work responsibilities (p13)<br><br>Innovative solutions to problems (p14 and 16)<br><br>Interesting but challenging time professionally (p14)	
40	Lack of PPE and confusion at the start caused tensions at work. Was working despite workplace identifying vulnerable groups and her being in a vulnerable demographic due to the lack of non-vulnerable staff. * (not sure if this is reportable as immunocompromised HCW would be identifiable if she is the only one with this theme) P12		Access to services was limited and could not access counselling for strained relationship with husband. P7
41	Debriefing with colleagues<br><br>Org could do more to provide clarity/assurance p15<br><br>More workplace collegial and self-care support, trying to do together things apart p16.<br><br>Sense of support from mngt re selfcare and family needs, p 17	Exhaustion due to PPE and changing ED presentations (more MH,	Daughter struggling with homeschooling, routine and MH, found a study buddy for LD2 p9<br><br>Change in parenting – as going out less, not worried about where they are/who they are with, but about social/emotional wellbeing more…p13<br><br>Remaiming positive FHW role helps keep focus on why we are doing this, p13,<br><br>Limited information/news and just did what she could control, p13<br><br>Self-care, gardening offers time out p 15
42		Workplace changes were stressful- Adapting to Telehealth, home assessments/visits P. 9,10<br><br>Workplace enforcement of rules were very stressful- Mismatch between org rules/decisions and participant's choices/preferences P.9,10,11	Working more because of financial pressure.<br>P .3<br><br>Less activities-positive-more awareness of issues faced by family. P 20,21<br><br>Maintaining routines was important P.5,20<br><br>Experienced difficulties during the initial stages P.6,7<br><br>Child had difficulties adapting to lockdown environment-Impacted mood, resistant to rules, academic impacts.<br>P 14, 17, 19, 20
43	Initial fear, not a lot of direction/ support, had to make it up – didn't feel risks were being managed. Now more org support, better trained and equipped, and stable/reduced community transmission.	Lots of change/pressure at work, good to come home, have things stable p8	Some censoring amount of info so they weren't too scared, p5<br><br>Enviro impact of PPE p10

ID	Workplace support	Work routines/ procedures	OTHER
1*	Working long hours (p14) but no sense of annoyance with it<br><br>Early access to PPE a problem but organisation communicated and did what they could about it (p16)<br><br>Positive work supports and examples of maternity and social work giving self-care suggestions (innovative) (p18)	Change in work responsibilities (p13)<br><br>Innovative solutions to problems (p14 and 16)<br><br>Interesting but challenging time professionally (p14)	
	Managing fear for team – had to train, talk through what was effective, risk avoidant for daughter, kept her home P11<br><br>Strategies from work – reduce focus on KPI, so more time to manage PPE, counselling (now, not initially), lots of extra meetings to communicate. P12<br><br>Different managers had different approaches to WfH – her manager quite suspicious, had to justify it, others encouraged to be home. Suspicion indicated lack of trust: relaxed over time.<br>Initial optional re home schooling etc., then workplace couldn't manage social distancing, so as it evolved, motivations changed<br>p12	Change in own prep/set up – would wear make up etc – now focus on comfort (breathable clothes etc)< because v sweaty in gowns…p10	
44	Previously felt unsupported by workplace in parenting (before adding pressure of COVID) – p 7, 12,13, 15<br><br>Pre-existing tensions between work/parenting demands exacerbated during COVID – p. 7<br><br>No one to debrief with – and no social support for this – p. 24	Keeping head around changes p.2, 6, 26<br><br>Juggling demands of work and home p. 2<br><br>Already adapting to changes at work before COVID – p. 3<br><br>Difficulty juggling home and work even before COVID – p. 3-4<br><br>Increased caseload – p 4<br><br>Many workplace changes – p. 4, 5<br><br>Limited resource/computer availability p. 4.<br><br>Difficulties of working in PPE – p. 5<br><br>Dong extra hours to keep up with changes – p. 6<br><br>Work pressure impacting on relationship with son – p6<br><br>fast pace of workplace changes – p. 25	

ID	Workplace support	Work routines/ procedures	OTHER
1*	Working long hours (p14) but no sense of annoyance with it<br><br>Early access to PPE a problem but organisation communicated and did what they could about it (p16)<br><br>Positive work supports and examples of maternity and social work giving self-care suggestions (innovative) (p18)	Change in work responsibilities (p13)<br><br>Innovative solutions to problems (p14 and 16)<br><br>Interesting but challenging time professionally (p14)	
		Handovers, hotline and case conferences have helped with changes – p 26	
45		Routines and response to infection control changed as outbreak changed (p.17)	Family struggles with transition post lockdown and children's worries (p.9, 10)
46	Also safety concerns – if in scrubs etc, being abused in shops or getting coffees even if on the way to a shift, so clean. Directive from work to get changed to stay safe and minimise infection risk. P2<br><br>Felt there was more support internationally, but locally being abused, not feeling safe.p2<br><br>Missed medical colleague debriefing – used husband but not the same…p3, p5<br><br>Increase in MH presentations bigger concern than PPE /illness. Increased violence at work (Code Grey/Black are Occ Violence/Personal threats), husband was upset about, angry that they weren't keeping her safer. P5<br><br>Didn't feel there was great exec support p10<br><br>Did feel some safety at work – felt more infections came from community into work, Woolworths was more of risk. If contracted in community, would be quarantining as a family. P12-13<br><br>Permanently changed practices, always in mask now p13<br><br>Valued comms/debriefing with peers p 13<br><br>Felt reliant on others, didn't like lack of control p 14<br><br>Wanted more support from mngt, esp more MH support given presentations p15<br><br>Workplace support for families – offered accommodation/financial support, advise to change at work etc, but outside those, not really considering impact on families. P15	Lots of changes at work – have been an ANUM for over a decade, but changes in policy/emails each shift, so hard to stay across all the changes and ensure staff were up to date. P10-11, esp feeling respomnsible as a manager for staff.	

ID	Workplace support	Work routines/ procedures	OTHER
1*	Working long hours (p14) but no sense of annoyance with it<br><br>Early access to PPE a problem but organisation communicated and did what they could about it (p16)<br><br>Positive work supports and examples of maternity and social work giving self-care suggestions (innovative) (p18)	Change in work responsibilities (p13)<br><br>Innovative solutions to problems (p14 and 16)<br><br>Interesting but challenging time professionally (p14)	
51	Work supported clothing, but some other suggestions not realistic/practical, and/or tech options not available, Felt like different model in diff hospitals with regard to tech support/wfh…<br>Still felt strong requirement to deliver services, and doesn't stop with the pandemic p13-14<br><br>Didn't feel heard in terms of concerns at work p14<br><br>Clarity re procedures for AH teams not as good as for nursing/medical, and not practical given work and small team p15<br><br>Inconsistency in procedures between interactions with staff and patients, diff wards/campuses p15<br><br>Missed the collegial support p15-16<br><br>More tolerance for sick leave p16	Changing protocols to manage childcare/permits etc p2<br><br>Stress at work could flow into home, esp during peak # cases p10-11<br><br>Clarity around responses was different in diff parts of the organisation p 14<br><br>Initially naïve to what it all meant, then so many changes p17	I think the masks have made all of our jobs really difficult. I don't know if you can see, but like I made some of these which is like a, you know, who we are under the mask. Because, you know, a lot of the time, like at our role and most of the healthcare workers, we're looking for other body language and facial expressions on people to gauge their reactions. P17
53	Support from colleagues…The routine and social aspect of work helped – less isolated (p.1, 4, 8, 13)<br><br>Importance of planning day to manage demands and asking for help (p.13)<br><br>…noted that participant had to come up with suggestions/ideas to cope herself (p.13, 14)	Sudden changes – no time to prepare (p.4)<br><br>Practical side of role – juggling demands was challenging (p.12)<br><br>Work/family interface<br>…Changed hours – extra processes led to leaving work late (p.5)<br>…fatigue from PPE (p.6)	Mental health and other impacts on children (p.2, 3)
54	Previously, would enter ED casually, advocate for role, then was almost – only going in for a genuine specific clinical need, then when re-opening, go back in, and that felt uncomfortable…then a few weeks later, lots more cases, don't go back in p7<br><br>Used Mum to debrief<br><br>WfH was a huge process, lots of hoops so never bothered, although this was offered for some staff. Otherwise, just reminders re PPE. No consideration of family role from HCW employer. Comparison against NGO and husband's work were much more supportive, e.g. days not as a/leave (wellbeing days), UberEats vouchers, dropped off goodies including things for family, taking care of employees. P21	Wanted faster org response initially, hard with constant changes in advice re what to wear, where to go –led to concern - What am I doing now that in a few weeks will be seen as risky? P7<br><br>Sense of every day processes much more complex – have to sign in and declare every day was intimidating p22<br><br>Fatigue of keeping up with changes p23	Didn't talk about concerns re infection a lot as didn't want to frighten him, and kids are around. Was more able to talk to Mum because in a separate location, not exposing kids to that conversation… Was also helpful to debrief p8<br><br>Conscious of grandma's need for social engagement p10<br><br>Toddle – hard to understand the reasons why p13, struggled with why everyone else was busy p17<br><br>He was better at prioritising self-care, she didn't feel that she had time to engage in that (but prob didn't do it before hand either) p18, p19

ID	Workplace support	Work routines/ procedures	OTHER
1*	Working long hours (p14) but no sense of annoyance with it<br><br>Early access to PPE a problem but organisation communicated and did what they could about it (p16)<br><br>Positive work supports and examples of maternity and social work giving self-care suggestions (innovative) (p18)	Change in work responsibilities (p13)<br><br>Innovative solutions to problems (p14 and 16)<br><br>Interesting but challenging time professionally (p14)	
			Reflection on strengths – staying together, modelling that we can overcome stress, it was challenging but was positive to go through it together. Kids able to reflect see challenges for their sister, see how someone else is finding it hard. p24
55	Exhausted due to workplace changes (around IT etc) p. 17	Too many changes at work (see workplace support theme) p. 17<br><br>Changing schedules at work impacted on home life p. 5<br><br>Able to work from home but in secret so that other workers didn't know p 5	Family were able to reframe the situation by looking at how other countries coped/didn't cope p. 9 ,<br>p. 21 – continue to have fun
56**	Moved from a private to a public hospital for better and safer management of risk (p4)<br><br>Sense of betrayal from broader agencies in not supporting healthcare workers (p10)<br><br>On N95 masks not being made available (p10, 11) "It's like sending soldiers out over the frontline without helmets or without bulletproof vests when they're available"<br><br>Concerns re some gaps in support (p25)	Distress at inaction or delayed changes in early workplace (throughout)<br><br>Increased need to support and manage junior staff (p24)<br><br>"And that's my unofficial role, often is to have autocratic leaders, directors who are unapproachable and to be one of the more approachable private advisors, and that continued, but it was amplified." (p25)	
57			
58	Providing support for colleagues was important but difficult P.7<br><br>Felt supported by superiors P 32,33<br><br>Workplace's response was seen as good and helped confidence P.11,32<br><br>PPE and infection control helped with her and family confidence as a frontline hcw<br><br>P. 2,4,31	Took on additional work to help colleagues P.7,	Family relied on her for care and emotional support P. 20,22,15<br><br>Felt supported by partner 2,13<br><br>Home schooling was a positive P. 4,5<br>Worried About family and friends' safety during covid P 19,20<br>Felt isolated from extended family P 6,9,30,
59	Org support in the beginning was not adequate. Higher management was pushed to the limit and needed more support as well. P 24, 25	Context. ICU & respiratory clinical physician and trainer. There was uncertainty and unknowns at the beginning of covid that led to additional	Made comparisons with overseas counterpart. Relied on them for information. Source of fear and confusion. P.5,16

ID	Workplace support	Work routines/ procedures	OTHER
1*	Working long hours (p14) but no sense of annoyance with it<br><br>Early access to PPE a problem but organisation communicated and did what they could about it (p16)<br><br>Positive work supports and examples of maternity and social work giving self-care suggestions (innovative) (p18)	Change in work responsibilities (p13)<br><br>Innovative solutions to problems (p14 and 16)<br><br>Interesting but challenging time professionally (p14)	
	Concerned about trainees risks and safety. Offered living space to children of trainees 16,17,23	demands at work. Mainly around prepping and planning. P 4, 5, 23<br><br>Difficult to manage high workload with spending time with son P 8,10<br><br>There were additional work roles that added to the high workload. Needed to manage boundaries with these at work. P24<br><br>Needed to balance work/life and draw boundaries. P 23, 25<br><br>WfH difficult with child at home. p6, 7,14,15	Felt that school care for essential workers during this time was inadequate .Concerned about child's care. 9<br><br>Extended family plays a large part of being social support for their family but social distancing makes it hard for extended family to support them. P 8,11,12<br><br>Emotional burden. Extended family required emotional support. Hard to provide while in lockdown and following social distancing. P 15,17,18,22
60	Juggling roles (p.1,2,4)<br>Parenting relay (p.24)	Constant hypervigilance and worry and fear (p.2, 6) feeling 'dirty' (p.9)	Mental preparation for change (p.5)<br>Anticipatory stress of the pandemic (p.5, 6)<br>Increased demands of role (p.7, 8)
61	Context: single mum. Had to take covid tests. Had to have the kids isolated with ex-partner. Slow results meant that she wasn't able to take her kids earlier. P 6<br><br>Workplace was informative and there was lot of changes to protocol, but participant felt informed and positive. Felt that information was clear P10.11		Positive about vaccine but hesitant. P 21
62P	More anxious at work in Phase 2 but set up well at work<br><br>Support at work – asking about how families were doing<br><br>Felt comfortable managing risk at work but there was a learning curve in it. Wellbeing a focus in his department which helped p14<br><br>Can't recall family specific support from the organization but there was some from colleagues p15	Changes in criteria and information, adapted	
62C			Context – 15yo male, 2 younger siblings, father is FHW, mother wfh (research)

ID	Workplace support	Work routines/ procedures	OTHER
1*	Working long hours (p14) but no sense of annoyance with it<br><br>Early access to PPE a problem but organisation communicated and did what they could about it (p16)<br><br>Positive work supports and examples of maternity and social work giving self-care suggestions (innovative) (p18)	Change in work responsibilities (p13)<br><br>Innovative solutions to problems (p14 and 16)<br><br>Interesting but challenging time professionally (p14)	
			Hardest parts were around engagement/enjoyment of school<br>Best – chess going online, making the most of experience (p10)
63	Suggested that workplaces could extend some supports offered to partners and family members (p.18)	Change in routines and procedures at work meant a longer and more tiring work day (p.5)<br><br>Extra pressures and stress on role due to PPE and COVID (p.16)	Worry about impact of working full time on children, reflecting on her own childhood (p.8)<br><br>Impact of not seeing grandparents on children and their bond (p.9)<br><br>Other impacts on daughter from being at home more, thinks her social development was impacted (p.11, 12)<br><br>Impact on children's growth from not being unwell over past year, benefit to physical health and development (p.12)<br><br>Participant opened up with interviewer "we have delved pretty deep there. I have talked to you more about it than I have talked to anyone else ever." (p.14)
